# Quadratus Lumborum Block as Sole, Homeostatic-Preserving Anesthetic for a Patient with Multiple System Atrophy Undergoing Open Inguinal Hernia Repair: A Case Report

**DOI:** 10.1155/2018/7161860

**Published:** 2018-06-28

**Authors:** M. D. Luca La Colla, R. M. D. Schroeder

**Affiliations:** Department of Anesthesiology, Duke University Medical Center, Durham, NC, USA

## Abstract

Quadratus Lumborum (QL) block has been successfully used for different abdominal procedures in the past. Multiple system atrophy (MSA) is a progressive neurodegenerative disorder characterized mainly by autonomic instability, motor impairment, and cognitive dysfunction. We report a case of a patient with MSA with a history of multiple episodes of unplanned admissions following outpatient minor surgical procedures under general anesthesia scheduled to undergo open inguinal hernia repair. In our patient, QL block was successfully used for surgical anesthesia and it resulted in hemodynamic stability and an opioid-free perioperative course.

## 1. Introduction

Quadratus Lumborum (QL) block was first described in 2007 by Blanco [1] and can provide reliable analgesia following different abdominal [2] as well as hip procedures [3, 4] and even amputations [5].

Multiple system atrophy (MSA) is a progressive neurodegenerative disorder characterized mainly by autonomic failure (including autonomic instability) and motor impairment. Anesthesia of any kind in affected patients carries significant risk [6]. Inguinal hernia repair (IHR) is one of the most commonly performed surgical procedures and is routinely performed under general, spinal, or even local anesthesia with sedation. All of these pose significant risk for patients afflicted with MSA.

To the best of our knowledge QL block has never been used as primary anesthetic choice for inguinal hernia repair in adults.

Written informed consent for both anesthesia and publication of patient's data in an anonymous form was obtained and documented in the patient's chart. The 2013 CARE checklist for writing case reports was followed.

## 2. Description of the Case

A 69-year-old male with a history of MSA and obstructive sleep apnea was scheduled for elective open repair of a symptomatic right inguinal hernia. In particular, he suffered from autonomic dysfunction, severe orthostatic hypotension, and cognitive impairment with several unplanned admissions following outpatient minor orthopedic surgical procedures due to “slowness to wake up” (according to his wife) or delirium requiring restraints after postoperative opioid administration.

Given his history, it was felt that avoiding general anesthesia was advisable. His severe orthostatic hypotension also made subarachnoid block potentially unsuitable considering the risk of hypotension. There was also concern that he would not be able to cooperate with local anesthesia performed by the surgeon without deep sedation. Considering the patient's prior history and comorbidities, a QL block was chosen as the sole anesthetic.

Written informed consent for both anesthesia and publication of the patient's data in an anonymous form was obtained and documented in the patient's chart. The 2013 CARE checklist for writing case reports was followed.

The patient was transferred to the preoperative holding area and ASA standard monitors were applied. He was placed in the supine position slightly tilted to the left to better expose the right flank and received fentanyl 50 mcg for sedation. After sterile preparation and using standard aseptic technique, a high-frequency linear array probe covered by sterile plastic sleeve was placed horizontally on the umbilicus and moved laterally. Both rectus abdominis and the 3 muscle layers of the abdominal wall were identified and traced posteriorly to the point where the deep fascia of the transversus abdominis merges with the thoracolumbar fascia. A 20-G 10-cm nonstimulating echogenic Tuohy needle was advanced in-plane with the probe until appropriate location for a QL type I block according to Blanco and McDonnell [1] was achieved. Needle positioning was confirmed by careful hydrodissection with normal saline and, after negative aspiration, 20 ml of 0.5% ropivacaine was injected and appropriate, posterior spread observed.

After a few minutes the patient was brought to the operating room and loss of pin-prick sensation was documented from T9 to L1 on the right side.

Prior to incision, the surgeon (unfamiliar with this block) decided to perform local infiltration along the incision site (no iliohypogastric and/or ilioinguinal block) with 6 mL of 0.25% bupivacaine. Intraoperative sedation was accomplished with low-dose propofol (10 mcg/kg/min). No additional parenteral opioids or sedative agents were administered. The patient's vital signs were stable and remained within 20% of his preoperative values. Of note, the patient did not require any additional supplementation of local anesthetic and the surgeon was pleasantly surprised.

Postoperative analgesia was supplemented by IV acetaminophen 1 g.

The patient was brought to the PACU awake, oriented, and pain-free, and he was discharged to the floor within 45 minutes. There was no evidence of delirium, agitation, or other issues during the perioperative period, and he was discharged home the following day after receiving 2 scheduled doses of acetaminophen 975 mg and pregabalin 100 mg overnight.

## 3. Discussion

Multiple System Atrophy is a group of neurodegenerative syndromes characterized by autonomic dysfunction, cerebellar abnormalities, corticospinal degeneration and Parkinson-like symptoms. MSA patients are at risk for hemodynamic instability (due to impairment of baroreceptor activity and sympathetic hypersensitivity), airway problems including laryngeal stridor, central sleep apnea (with increased risk of prolonged ventilation, reintubation and emergency tracheostomy following general anesthesia), and gastric aspiration. These patients are also at risk for delirium and/or agitation making cooperation with regional, neuraxial, and especially local anesthesia potentially problematic. While there are published reports of surgical procedures under single-shot spinal anesthesia [7], the general consensus among practitioners seems to be that if possible, low-dose spinal or epidural anesthesia should be used and titrated carefully to minimize hemodynamic compromise [6]. Should general anesthesia be necessary, an arterial line should be placed [8].

Previous reports of reliable and widespread coverage with QL block [3, 4] led us to choose this block over the more popular TAP block. QL block has been shown to provide superior analgesia compared to TAP block after low abdominal surgery in children [9]. Scimia et al. [10] published a single case report of the use of an ultrasound-guided transversalis fascia block for a patient undergoing inguinal herniorrhaphy. According to some authors, though, the QL type 1 block (the approach we used in this case) and the transversalis fascia block are essentially the same block because the patterns of spread of the injectate are similar [11]. Therefore, Scimia et al. might have performed a QL-1 block.

In our opinion, the better coverage obtained by the QL-1 block compared to the traditional TAP block is due to more reliable block of the iliohypogastric/ilioinguinal as well as genitofemoral nerves in the posterior pararenal space. Even though prospective comparisons between QL and TAP block in adults are currently ongoing, preliminary data from a study in children show that QL provides superior and longer-lasting analgesia than TAP block [9]. For these reasons it was felt that QL-1 was a better choice than TAP block, general, and even spinal anesthesia and this approach allowed us to avoid arterial line placement for a minor surgical procedure. The QL block provided excellent surgical anesthesia with a single needle pass, caused as minimal if any perturbations in autonomic tone, and posed no risk to our patient's vocal cord function. Finally, it allowed him to remain as awake and oriented as possible, thus minimizing the risk of drug-induced delirium either intra- or postoperatively. The patient and his family, who had been far more concerned about anesthetic than surgical risk, were extremely pleased with the result.

In summary, we report the first use of QL type 1 block which provided excellent and complete surgical anesthesia for open inguinal repair in a patient with MSA, preventing further interventions that would likely have caused hemodynamic compromise or mental status changes. Even though further studies are necessary, this case report suggests that options other than general and spinal anesthesia are available and may be preferable in patients with MSA undergoing abdominal surgical procedures.
