# Erratum to “Quadratus Lumborum Block as Sole, Homeostatic-Preserving Anesthetic for a Patient with Multiple System Atrophy Undergoing Open Inguinal Hernia Repair: A Case Report”

**DOI:** 10.1155/2018/8316750

**Published:** 2018-08-19

**Authors:** Luca La Colla, Rebecca Schroeder

**Affiliations:** Department of Anesthesiology, Duke University Medical Center, Durham, NC, USA

In the article titled “Quadratus Lumborum Block as Sole, Homeostatic-Preserving Anesthetic for a Patient with Multiple System Atrophy Undergoing Open Inguinal Hernia Repair: A Case Report” [1], the names of the authors were given incorrectly as M. D. Luca La Colla and R. M. D. Schroeder. The authors' names should have been written as Luca La Colla and Rebecca Schroeder. The revised authors' list is shown above.
